# A Validated Chiral LC–MS/MS Method for the Enantioselective Determination of (S)-(+)- and (R)-(-)-Ibuprofen in Dog Plasma: Its Application to a Pharmacokinetic Study

**DOI:** 10.3390/pharmaceutics15030824

**Published:** 2023-03-02

**Authors:** Sanghee Choi, Wang-Seob Shim, Jiyoung Yoon, Doowon Choi, Jinseong Lee, Soo-Heui Paik, Eun-Kyoung Chung, Kyung-Tae Lee

**Affiliations:** 1 Department of Biomedical and Pharmaceutical Sciences, Graduate School, Kyung Hee University, Seoul 02447, Republic of Korea; 2 Kyung Hee Drug Analysis Center, College of Pharmacy, Kyung Hee University, Seoul 02447, Republic of Korea; 3 Department of BD&RA Division, BNC KOREA Inc., Seoul 06296, Republic of Korea; 4 College of Pharmacy, Sunchon National University, Suncheon-si 57922, Republic of Korea; 5 Department of Pharmacy, College of Pharmacy, Kyung Hee University, Seoul 02447, Republic of Korea; 6 Department of Pharmaceutical Biochemistry, College of Pharmacy, Kyung Hee University, Seoul 02447, Republic of Korea

**Keywords:** ibuprofen, dexibuprofen, enantiomer, LC–MS/MS, beagle dog plasma, validation, bioanalytical method validation, pharmacokinetics

## Abstract

The purpose of this study was to develop a method for simultaneously separating ibuprofen enantiomers using electrospray ionization (ESI) liquid chromatography with tandem mass spectrometry (LC–MS/MS). LC–MS/MS was operated with negative ionization and multiple reaction monitoring modes; transitions were monitored at *m*/*z* of 205.1 > 160.9 for ibuprofen enantiomers, 208.1 > 163.9 for (S)-(+)-ibuprofen-d3 [internal standard 1 (IS1)], and 253.1 > 208.9 for (S)-(+)-ketoprofen (IS2), respectively. In a one-step liquid–liquid extraction, 10 μL plasma was extracted with ethyl acetate:methyl tertiary-butyl ether of 7:3. Enantiomer chromatographic separation was carried out with an isocratic mobile phase consisting of 0.008% formic acid in water–methanol (*v*/*v*) at a flow rate of 0.4 mL/min on a CHIRALCEL^®^ OJ-3R column (150 × 4.6 mm, 3 µm). This method was fully validated for each enantiomer and results were in compliance with the regulatory guidelines of the U.S. Food and Drug Administration and the Korea Ministry of Food and Drug Safety. The validated assay was executed for nonclinical pharmacokinetic studies after oral and intravenous administration of racemic ibuprofen and dexibuprofen in beagle dogs.

## 1. Introduction

Ibuprofen, 2-(RS)-[4-(2-methyl propyl) phenyl] propionic acid, is a non-steroidal anti-inflammatory drug (NSAID) introduced in England in 1969. It is a commonly used NSAID with the lowest toxicity that plays an important role in the management of pain, inflammation, and fever owing to its inhibitory effect on cyclooxygenase-1 (COX-1) and cyclooxygenase-2 (COX-2), which are elaborated in prostaglandin synthesis [1,2,3,4]. It is mainly used as a racemic mixture comprising equal quantities of dexibuprofen [(2S)-2-(4-isobutylphenyl) propionic acid, (S)-(+)-ibuprofen] and (R)-(-)-ibuprofen [5]. (S)-(+)-Ibuprofen is the primary active isomer with pharmacological effects and R-(-)-ibuprofen is an inactive isomer; (S)-(+)-ibuprofen can inhibit cyclooxygenase (COX) at clinically relevant concentrations, but R-(-)-ibuprofen is not a COX inhibitor [6]. [(S)-(+)-ibuprofen], an ibuprofen enantiomer with pharmacological activity, was first launched in Austria in 1994 (Seractil^TM^) as a film-coated tablet [7]. Racemic ibuprofen and (S)-(+)-ibuprofen differ in their pharmacological properties, toxicity, water solubility, melting point, stability, and bioavailability [8,9,10]. On average, 63% of the administered dose of R-(-)-ibuprofen is stereospecifically converted to (S)-(+)-ibuprofen in humans [11]. However, (S)-(+)-ibuprofen is not reversed to R-(-)-ibuprofen; only a unidirectional reversal of R-(-)-ibuprofen to (S)-(+)-ibuprofen occurs, and the in vivo metabolism of its carboxyl metabolite is not stereoselective [6,11,12,13,14]. A previous study suggested that (S)-(+)-ibuprofen is the preferred substrate for both phase I and II metabolism compared to R-(-)-ibuprofen [15]. (S)-(+)-Ibuprofen is preferentially metabolized by CYP2C9 and (R)-(-)-Ibuprofen by CYP2C8 [15,16,17]. According to a previous study, the isomeric conversion after racemate is 35–85% and varies depending on the formulation type, disease state, individual characteristics, and species [9,11,18]. (S)-(+)-Ibuprofen has the advantages of lower toxicity, greater pharmacological activity, and less variation in clinical efficacy compared to racemic ibuprofen. (S)-(+)-Ibuprofen has been developed as oral formulations, eye drops, and transdermal patches [9]. Intravenous ibuprofen is also used to treat patent ductus arteriosus in neonates, control acute and severe pain after surgery, and reduce the use of narcotic opioids when managing pain [19]. However, to our knowledge, no published pharmacokinetic reports of the intravenous injection of (S)-(+)-ibuprofen have been quantified using an enantioselective LC–MS/MS method.

Several bioanalytical methods have been developed to quantify ibuprofen in biological samples, including high-performance liquid chromatography (HPLC) with ultraviolet (UV) or fluorescence [20,21,22,23,24,25,26,27,28,29,30], gas chromatography (GC) [31], and gas chromatography–mass spectrometry (GC–MS) [32,33,34]. Although HPLC is commonly used, there are limitations to this method, such as the large sample volume (100–500 µL plasma) required for bioanalysis [20,21,22,23,24,25,26,27,28,29,30] and lack of stereoselectivity [20,21]. Previously developed stereoselective HPLC with fluorescence detection after derivatization involves complicated processing procedures, limiting its general applicability [26,27,28,29,30]. GC and GC–MS methods using derivatization require prolonged pretreatment time [33,34] and have low (or insufficient) sensitivity (5–15 µg/mL) [31,32] and inadequate stereoselectivity [31,32,34]. Mass spectrometers are good alternatives for overcoming these limitations owing to their high throughput, sensitivity, and selectivity [35,36,37]. Indeed, several previously developed liquid chromatography methods with tandem mass spectrometry detection (LC–MS/MS) are available for the analysis of ibuprofen enantiomers in biological samples [38,39,40,41,42,43]. Nevertheless, most of these methods show comparably low sensitivity (limit of detection, 20–50 ng/mL) [38,41,42], insufficient recovery [38,41,43], and require a large sample volume (100–500 µL of plasma) for analysis [39,40,42,43]. An indirect method employing derivatization to form diastereomers with LC–MS/MS has a short run time (5 min). However, the pretreatment process is complex and requires a longer pretreatment time [39,43]. Thus, our objective was to develop a sensitive, selective, reproducible, efficient, and sound LC–MS/MS design for high-throughput stereoselective analysis of (S)-(+)-ibuprofen. The bioanalytical method developed in this study was fully validated and successfully applied to nonclinical pharmacokinetic studies in beagle dogs receiving oral and intravenous racemic ibuprofen and (S)-(+)-ibuprofen.

## 2. Materials and Methods

### 2.1. Chemicals and Reagents

(S)-(+)-Ibuprofen (purity 99.74%), (R)-(-)-Ibuprofen (purity 99.89%), and (S)-(+)-ibuprofen-d3 [internal standard (IS1), purity 98.0%, isotopic 99.6%)] were purchased from TRC (Toronto, ON, Canada). (S)-(+)-Ketoprofen [internal standard (IS2), purity 99.9%] and formic acid were purchased from Sigma-Aldrich (St. Louis, MO, USA). HPLC-grade methanol, ethyl acetate (EtOAc), and methyl tertiary-butyl ether (MTBE) were purchased from Baker (Phillipsburg, NJ, USA). For the HPLC analysis, tertiary purified water (DW) was obtained using a Milli-Q^®^ water purification system (Millipore Co., Billerica, MA, USA). HPLC-grade chemicals and solvents were used in the HPLC analyses.

### 2.2. LC–MS/MS System

Liquid chromatography was performed on an Agilent 1200 Series HPLC system (Agilent, Santa Clara, CA, USA), and chromatographic separation was carried out using a CHIRALCEL^®^ OJ–3R column (150 × 4.6 mm, 3 µm) (Daicel Corporation, Osaka, Japan). The mobile phase consisted of 0.008% formic acid in 85% methanol (MeOH/DW = 85:15, *v*/*v*) and was pumped at a flow rate of 0.4 mL/min. Mass spectrometry was operated on an API 4000 triple-quadrupole mass spectrometer (AB SIEX, Framingham, MA, USA) equipped with an electrospray ion source. The optimized source parameters for (S)-(+)-ibuprofen, (R)-(-)-ibuprofen, IS1, and IS2 are listed in Table 1. Analytical data were handled using Analyst^®^ 1.6.2. (AB SCIEX, Framingham, MA, USA).

### 2.3. Preparation of Calibration Standards and Quality Control (QC) Samples

Stock solutions containing 1 mg/mL (S)-(+)-ibuprofen, (R)-(-)-ibuprofen, IS1, and IS2 were made in 100% methanol. The working solutions were diluted with 50% methanol (*v*/*v*), and all stock and working solutions were stored in a freezer at 20 °C. Calibration standards were processed by spiking plasma with (S)-(+)-ibuprofen and (R)-(-)-ibuprofen at 0.1, 0.5, 2, 10, 20, 40, and 80 µg/mL. Quality control samples were diluted with 50% methanol (*v*/*v*) using other stock solutions to obtain low, medium, and high concentrations of 0.3, 30, and 64 µg/mL. Solutions containing 10 µg/mL IS1 and IS2 were prepared in 50% methanol.

### 2.4. Plasma Sample Preparation

Plasma samples treated with heparin were thawed at room temperature on the day of analysis. Each plasma sample (10 μL) was placed in a microtubule. IS1 (20 μL, 10 µg/mL), IS2 (20 μL, 10 µg/mL), 0.1% formic acid (50 μL), and 1 mL of EtOAc:MTBE = 7:3 (*v*/*v*) were added to each microtube. After vortexing for 5 min and centrifuging at 20,800 *g* for 10 min, the supernatant (1.0 mL) was moved to a clean microtube, evaporated, and dried under N_2_ gas at 40 °C. The residues were reconstituted in 100 μL of 50% methanol. For analysis, 10 μL of the supernatant was injected into the analytical column.

### 2.5. Method Validation

The developed bioanalytical method was validated in terms of selectivity, lower limits of quantification, linearity, precision, accuracy, recovery, matrix effect, stability, carryover, and dilution integrity. Bioanalytical assay validation was accomplished according to the method validation guidelines of the U.S. Food and Drug Administration (USFDA) and the Ministry of Food and Drug Safety in Korea (MFDS) [44,45].

#### 2.5.1. Selectivity and Sensitivity

Selectivity is a method for determining whether the analyte and IS are quantifiable separately from the other substances present in the sample. Selectivity was determined by analyzing seven randomly selected blank beagle dog plasma samples. Method selectivity and sensitivity were evaluated by comparing chromatograms of blank plasma, plasma spiked with IS1 (10 μg/mL) or IS2 (10 μg/mL), plasma spiked with (S)-(+)-ibuprofen (80 μg/mL) or (R)-(-)-ibuprofen (80 μg/mL), and plasma spiked with (S)-(+)-ibuprofen (0.1 μg/mL), (R)-(-)-ibuprofen (0.1 μg/mL), IS1(10 μg/mL), and IS2 (10 μg/mL). The lower limit of quantification (LLOQ) was described as the lowest concentration of the calibration curve with a signal-to-noise ratio (S/N) greater than 10. The limit of detection (LOD) was described as the lowest concentration of mass detected with an S/N ratio greater than 3.

#### 2.5.2. Linearity and Carryover

Calibration curves of the standards were composed at seven (S)-(+)-ibuprofen and (R)-(-)-ibuprofen concentrations (0.1, 0.5, 2, 10, 20, 40, and 80 µg/mL). Linearity was determined by the calculated peak area ratios (*x*) of the standard to IS versus the concentrations of the standard (*y*) using weighted (1/*x*^2^) linear least squares regression (*y* = a*x* + b). A calibration curve with a correlation coefficient (*r*^2^) of 0.99 or greater was considered adequate. Carryover evaluates whether the residual analyte of the previously injected sample before the analysis affects the injection of the next sample during continuous measurement of the sample. Carryover was evaluated by injecting a blank sample after injecting an upper limit of quantification (ULOQ) of the calibration curve standard sample.

#### 2.5.3. Precision and Accuracy

Inter- and intra-assays were performed using replicate analyses of validation sample concentrations (0.1, 0.3, 30, and 64 μg/mL) on three consecutive days. The mean and standard deviation (SD) of the concentrations calculated for these batches were estimated. Accuracy and precision were calculated as relative error and coefficient of variation (CV), respectively. The acceptance criterion was within ±15% of the nominal concentration, except for the LLOQ, which was within ±20%.

#### 2.5.4. Recovery and Matrix Effect

The recovery and matrix effects were evaluated at three QC concentrations and analyzed using analytes spiked before the pre-extraction matrix ([A]), analytes spiked after the post-extraction matrix ([B]), and pure analyte solutions in 50% methanol ([C]). The recoveries of (S)-(+)-ibuprofen, (R)-(-)-ibuprofen, and ISs at QC concentrations were evaluated using the ratio (A/B × 100)%. The matrix effect of (S)-(+)-ibuprofen, (R)-(-)-ibuprofen, IS1, and IS2 associated with ion suppression or enhancement caused by the plasma matrix was assessed using the ratio (B/C × 100)%.

#### 2.5.5. Stability

The stability of the stock and working solutions was evaluated in triplicate for low and high QC concentrations stored at room temperature for 3 and 7 h, respectively, by comparing their peak areas with those of freshly prepared stock and working solutions. The stability of (S)-(+)-ibuprofen and (R)-(-)-ibuprofen in plasma was evaluated by analyzing samples in triplicate for each QC concentration of 0.3, 30, and 64 μg/mL under the following experimental conditions: freeze–thaw stability after three freeze–thaw cycles at −70 °C; short-term stability at room temperature, 4 °C, and −70 °C for 7 h; auto-sampler stability at 10 °C for 54 h; and long-term stability at −70 °C for 196 days.

#### 2.5.6. Dilution Integrity

Dilution integrity was investigated for samples with quantified concentrations outside the calibration curve range. Beagle dog plasma samples with (S)-(+)-ibuprofen and (R)-(-)-ibuprofen concentrations above the ULOQ (80 μg/mL) were diluted with blank beagle dog plasma and reanalyzed. Diluted QC samples of (S)-(+)-ibuprofen and (R)-(-)-ibuprofen, which had 5-fold higher concentrations than those of the QC 0.3, 30, and 64 μg/mL, were prepared as described in Section 2.3. The diluted QC samples were diluted five times with blank beagle dog plasma at the original QC concentration and preparation, as described in Section 2.4. Precision and accuracy were assessed in terms of CV (acceptable range: <15%) and RE (acceptable range: within ±15%), respectively.

### 2.6. Application to a Pharmacokinetic Study

The newly developed quantitation method was executed to analyze plasma samples obtained in a nonclinical pharmacokinetic study after oral and intravenous administration of (S)-(+)-ibuprofen and racemic-ibuprofen to healthy male beagle dogs (*n* = 12) weighing 8.68 ± 0.29 kg (Oriental bio Co., Ltd., Gapyeong, Republic of Korea). The animals were maintained under standardized conditions and fasted for 16 h before ibuprofen administration. In the pharmacokinetic study, these beagle dogs were randomly assigned to one of four treatment groups (*n* = 3 per group): racemic ibuprofen 400 mg administered as an oral tablet (Group 1: Brufen Tablet^®^, Samil-Pharm Ltd., Gyeonggi-do, Republic of Korea) and intravenous injection (Group 2: Amoburofen Injection^®^, Huons Ltd., Chungcheongbuk-do, Republic of Korea), and 300 mg (S)-(+)-ibuprofen administered as an oral tablet (Group 3: Ronofen Tablet^®^, TaiGuk Pharm Ltd., Gyeonggi-do, Republic of Korea) and intravenous injection (Group 4, BNC Korea Ltd. Sejong-si, Republic of Korea). Blood samples (2.0 mL) were drawn from the jugular vein at the following time points: 0 (before oral administration); 10, 20, and 40 min and 1, 1.5, 2, 2.5, 4, 6, 8, and 12 h (after oral administration); 0 (before intravenous administration); and 5, 10, 20, and 30 min and 1, 1.5, 2.5, 4, 6, 8, and 12 h after intravenous administration. Blood samples were collected in heparin-treated tubes (5 IU/mL). The blood samples were centrifuged immediately, and the plasma was separated and stored at −70 °C until LC–MS/MS analysis was executed. All study procedures were performed in compliance with the Animal Experimental Ethics Regulations of Notus Co., Ltd. (Guri, Korea, KNOTUS IACUC, protocol code: 21–KE-571; date of approval: 11 August 2021).

Pharmacokinetic parameters including extrapolated AUC_inf_ (area under the plasma concentration–time curve from time zero to infinity), AUC_last_ (area under the plasma concentration–time curve from time zero to the time of the last measurable concentration), *C*_max_ (maximum plasma drug concentration), *T*_max_ (the time to reach *C*_max_), terminal half-life (t_1/2_), apparent total clearance of the drug from plasma after oral administration (CL/F), apparent volume of distribution during the terminal phase after oral administration (V_z_/F), and mean residence time (MRT) were determined using noncompartmental methods (WinNonlin version 8.1; Pharsight Corporation, Mountain View, CA, USA).

### 2.7. Incurred Sample Reanalysis (ISR)

ISR was performed via computerized selection (sampling without replacement) of 15 subject samples near *C*_max_ and at the elimination phase of the pharmacokinetic profile. The results were compared to the initial data obtained from the same samples using the same procedure. The percentage change in the measured values did not exceed ± 20%.

## 3. Results and Discussion

### 3.1. Method Development

#### 3.1.1. Mass Spectrometry

**Figure 1:**
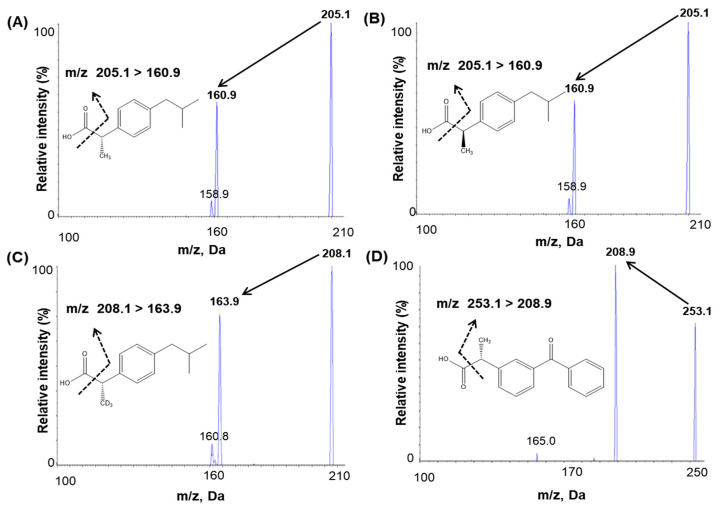
Product ion mass spectra and the fragmentation of (**A**) (S)-(+)-ibuprofen, (**B**) (R)-(-)-ibuprofen, (**C**) (S)-(+)-ibuprofen-d3 (internal standard, IS1), and (**D**) (S)-(+)-ketoprofen (internal standard, IS2).

An infusion was performed to optimize mass spectrometric conditions. For infusion, 100 ng/mL (S)-(+)-ibuprofen, (R)-(-)-ibuprofen, IS1, and IS2 solutions were injected into the mass spectrometer using a syringe pump at a flow rate of 10 μL/min. The maximum intensities of the product and fragment ions of ibuprofen and IS were obtained with an electrospray (ESI) negative interface because the negative mode of MS detection conditions was better ionized, more sensitive, and had no interference. Full-scan mass spectra were characterized by deprotonated molecules [M–H]^−^ at *m*/*z* 205.1 for (S)-(+)-ibuprofen and (R)-(-)-ibuprofen and *m*/*z* 208.1 for IS1 and *m*/*z* 253.1 for IS2. The selected product ion *m*/*z* were 160.9 for both (S)-(+)-ibuprofen and (R)-(-)-ibuprofen, 163.9 for IS1, and 208.9 for IS2 (Figure 1). The ionization source settings of the mass parameter according to the signal intensity were optimized by flow injection analysis tuning. The optimized mass parameters of the temperature and ion spray voltage were 450 °C and −4500 V, respectively, bringing about the highest signal intensity among the tested conditions. Chromatography revealed that analytes and IS fragments did not interfere with each other.

#### 3.1.2. Chromatographic Conditions

We achieved an appropriate peak shape, separation, and run time through developing the chromatographic conditions for separating ibuprofen enantiomers, including the columns, column temperature, mobile phase composition, and flow rate. In this study, the following reversed-phase columns and chiral columns were evaluated for favorable separation: DAICEL CHIRALPAK^®^ AGP (150 × 2.0 mm, 5 µm), DAICEL CHIRALCEL^®^ OJ–RH (150 × 4.6 mm, 5 µm), DAICEL CHIRALCEL^®^ IJ–3 (150 × 4.6 mm, 3µm), and DAICEL CHIRALCEL^®^ OJ–3R (150 × 4.6 mm, 3 µm). Among the tested columns, the DAICEL CHIRALCEL^®^ OJ–3R column exhibited the best results regarding peak shape, separation, sensitivity, and retention time. Chiral columns, such as α-acid glycoprotein (AGP), OJ–RH, and IJ–3 columns did not show complete separation, leading to inadequate peak shape and sensitivity. Mobile phases containing diverse buffers, such as formic acid and ammonium acetate, were evaluated using gradient and isocratic methods. The optimal column temperature and mobile phase flow rates were determined in various trials regarding peak sensitivity and chromatographic separation of enantiomers. The retention time of the peak was unstable when a gradient mobile phase was used with two different pumps. Therefore, as suggested in previous studies using the LC–MS/MS system [38,39,40,41,42], an isocratic mobile phase was used with the aqueous solvent mixed with the organic solvent to reduce the variation in the separation performance of the LC system. Among the tested solvents for the mobile phase, acetonitrile resulted in an excessively high baseline and delayed analyte elution. Methanol also exhibited a high baseline, but when formic acid buffer was added to the buffer, the baseline was considerably lowered and stabilized. Therefore, methanol was selected as the optimal organic solvent for the mobile phase, which was determined based on the peak shape and resolution. Depending on the concentration of formic acid, a different sensitivity and chiral separation by the interference of baseline was found. During the development of our method using various concentrations of formic acid, the optimal concentration of formic acid was determined to be 0.008% for achieving both chiral separation and sensitivity. The best separation results were obtained with the OJ–3R column at a temperature of 40 °C using an isocratic mobile phase constituting 85% methanol (water:methanol = 15:85, *v*/*v*) with 0.008% formic acid at a flow rate of 0.4 mL/min. Overall, the optimized chromatographic conditions mentioned above achieved the best peak shape, separability, sensitivity, and reproducibility.

#### 3.1.3. Sample Preparation

Various methods, such as solid-phase extraction (SPE), (liquid–liquid extraction) LLE, and derivatization, have been used to extract ibuprofen [38,39,40,41,42,43]. A previous study analyzing ibuprofen enantiomers found lower matrix effects with LLE than with SPE when extracting ibuprofen from biological samples [41]. In addition, LLE is a simpler and faster extraction method than the derivatization or SPE methods; therefore, a simple, one-step LLE extraction was utilized without the derivatization process as the extraction method in our study. Various conditions were tested to obtain better recovery rates and sensitivities using less plasma than previously reported methods [38,39,40,41,42,43]. To determine the optimal extraction conditions with respect to recovery and matrix effects, various extraction solvents were evaluated along with methylene chloride, EtOAc, *n*-hexane, MTBE, and mixtures of MTBE:EtOAc (8:2, 7:3, 3:7, 2:8; *v*/*v*) and EtOAc:*n*-hexane (8:2, 7:3, 3:7, 2:8; *v*/*v*). Extraction buffers, such as acetic acid, formic acid, hydrochloric acid, and reconstitution solvents, including methanol and acetonitrile, were assessed to achieve the optimal analyte peak shape and intensity. The best peak intensity and analyte recovery were obtained using 1 mL of MTBE:EtOAc (3:7; *v*/*v*) with 50 μL of 0.1% formic acid buffer as the extraction solvent and 50% methanol as the reconstitution solvent in our present study. Therefore, the developed plasma sample extraction method is a fast and simple extraction procedure with good sensitivity using a small amount of plasma (10 μL); thus, an abundant number of plasma samples can be analyzed.

### 3.2. Method Validation

#### 3.2.1. Selectivity and Sensitivity

**Figure 2:**
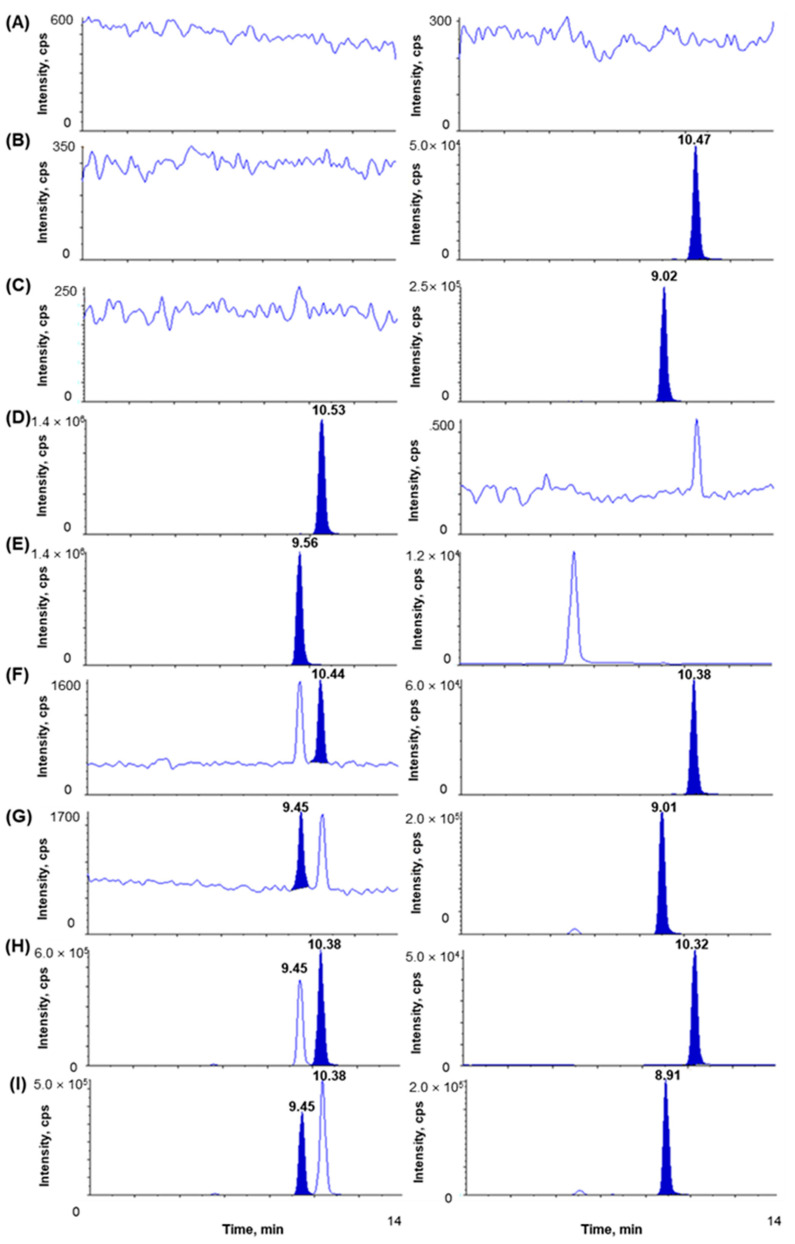
Chromatograms of (**A**) blank dog plasma, (**B**) plasma spiked with IS1 (10 µg/mL), (**C**) plasma spiked with IS2 (10 µg/mL), (**D**) plasma spiked with (S)-(+)-ibuprofen only (80 µg/mL, ULOQ), (**E**) plasma spiked with (R)-(-)-ibuprofen only (80 µg/mL, ULOQ), (**F**) plasma spiked with (S)-(+)-ibuprofen (0.1 µg/mL) and IS1 (10 µg/mL), (**G**) plasma spiked with (R)-(-)-ibuprofen (0.1 µg/mL) and IS2 (10 µg/mL), (**H**) dog plasma sample at 1 h after oral administration of racemic ibuprofen 400 mg tablet ((S)-(+)-ibuprofen measured concentration 75.96 µg/mL), (**I**) dog plasma sample at 1 h after oral administration of racemic ibuprofen 400 mg tablet ((R)-(-)-ibuprofen measured concentration 52.99 µg/mL). Boards on the right side are for IS.

**Figure 3:**
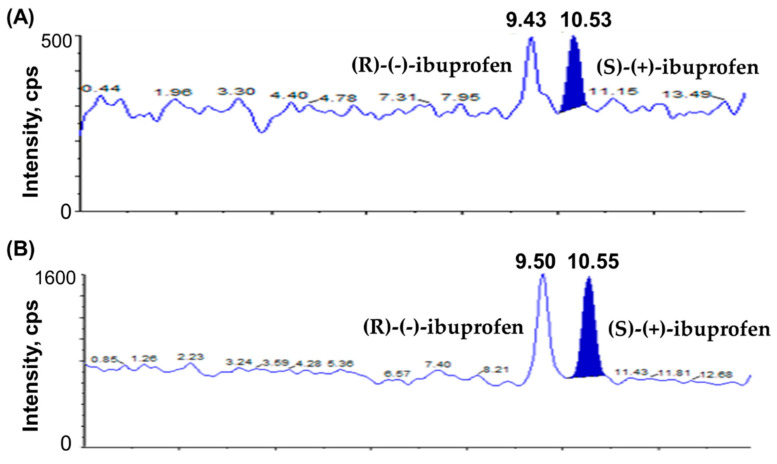
Chromatograms of (**A**) LOD of ibuprofen enantiomers ((S)-(+)-ibuprofen and (R)-(-)-ibuprofen) at 0.01 µg/mL and (**B**) LLOQ of ibuprofen enantiomers ((S)-(+)-ibuprofen and (R)-(-)-ibuprofen) at 0.1 µg/mL.

The chromatograms of blank plasma; plasma spiked with IS1 (10 µg/mL); plasma spiked with IS2 (10 µg/mL); plasma spiked with (S)-(+)-ibuprofen only (80 µg/mL); plasma spiked with (R)-(-)-ibuprofen only (80 µg/mL); plasma spiked with (S)-(+)-ibuprofen (0.1 µg/mL), (R)-(-)-ibuprofen (0.1 µg/mL), IS1 (10 µg/mL), and IS2 (10 µg/mL) show no interrupting peaks at the retention times of analytes and ISs, indicating acceptable selectivity of the newly developed analytical method (Figure 2). The LLOQ is 0.1 µg/mL for ibuprofen with signal-to-noise (S/N) ratios > 10, suggesting an adequately susceptible method to assess ibuprofen in plasma after the oral and intravenous administration of ibuprofen to beagle dogs. The LLOD is 0.01 µg/mL for ibuprofen with S/N ratios of >3. Chromatograms of the samples at the LLOQ and LLOD are shown in Figure 3. This newly developed method has lower LOD than previously reported methods [38,41,42].

#### 3.2.2. Linearity and Carryover

Considering the higher anticipated plasma concentrations after intravenous administration, we established a wider linearity range than that in previous reports [38,39,40,41,43]. All calibration curves for (S)-(+)-ibuprofen and (R)-(-)-ibuprofen demonstrated seven concentrations in the range of 0.1–80 µg/mL and exhibited linearity for dog plasma samples. The linear regression equation for the calibration curves (*n* = 5) was as follows: mean ± standard deviation (SD) of the slope and intercept: *y* = 0.152 (±0.005) *x* − 0.00677 (±0.000809) for (S)-(+)-ibuprofen (*r*^2^ ≥ 0.9979) and *y* = 0.0444 (±0.0029) *x* − 0.000302 (±0.000555) for (R)-(-)-ibuprofen (*r*^2^ ≥ 0.9940). The correlation coefficient was higher than 0.99 for all curves, and the CV of the slope was <15% in the analyzed concentration range. The linearity results over the calibration range of ibuprofen enantiomers are displayed in Table 2. No significant carryover was observed for (S)-(+)-ibuprofen or (R)-(-)-ibuprofen.

#### 3.2.3. Precision and Accuracy

Table 3 summarizes the intra- and inter-day precision and accuracy of (S)-(+)-ibuprofen and (R)-(-)-ibuprofen. The intra-day precision of the method for determining (S)-(+)-ibuprofen and (R)-(-)-ibuprofen ranged from 0.53 to 7.31% and from 0.48 to 7.19%, with accuracies ranging from 96.53 to 103.60% and 97.60 to 108.51%, respectively. The inter-day precision ranged from 1.28 to 6.60% and 1.04 to 4.87%, with accuracies ranging from 96.45 to 103.00% and 99.07 to 110.90% for measuring (S)-(+)-ibuprofen and (R)-(-)-ibuprofen concentrations, respectively. All results were within the ranges of precision (%) and accuracy (%) specified in the guidance of the MFDS and USFDA for bioanalytical applications [44,45].

#### 3.2.4. Recovery and Matrix Effect

To develop a bioanalytical method with excellent reproducibility, it is important to maximize recovery rates and minimize matrix effects [38,41,43]. Table 4 summarizes the results of the evaluation of the matrix effects and recovery rates. After preprocessing with the LLE method, the mean extraction recoveries of dog plasma at three QC concentrations (0.3, 30, and 64 µg/mL, *n* = 6) were 82.23–85.28% for (S)-(+)-ibuprofen and 84.01–87.89% for (R)-(-)-ibuprofen. The mean extraction recoveries for IS1 (10 µg/mL, *n* = 6) and IS2 (10 µg/mL, *n* = 6) were 87.49% and 84.56%, respectively. The estimated CV (%) values of the extraction recovery rates were within ± 15%, suggesting the high reproducibility of the sample preparation procedure.

The mean matrix effects for (S)-(+)-ibuprofen and (R)-(-)-ibuprofen at QC concentrations (0.3, 30, and 64 µg/mL, *n* = 6) were 86.74–93.79% and 94.38–100.99%, respectively. For IS1 (10 µg/mL, *n* = 6) and IS2 (10 µg/mL, *n* = 6), the mean matrix effects were 87.48% and 92.21%, respectively. The CV (%) values of the matrix effects were within ± 15%. Therefore, our newly developed bioanalytical assay had no considerable matrix effects between endogenous substances in dog plasma and analytes and no significant ion enhancement or suppression effects by (S)-(+)-ibuprofen or (R)-(-)-ibuprofen.

#### 3.2.5. Stability

The results of the stability study are represented in Table 5. Compared to a freshly prepared stock solution of (S)-(+)-ibuprofen and (R)-(-)-ibuprofen, the mean % peak area of the stock solution of (S)-(+)-ibuprofen and (R)-(-)-ibuprofen at room temperature for 3 h was 99.04%–100.72%; the mean % peak area of the working solution of (S)-(+)-ibuprofen and (R)-(-)-ibuprofen at room temperature for 7 h was 98.83%–101.73%. The mean stabilities of (S)-(+)-ibuprofen and (R)-(-)-ibuprofen in beagle dog plasma were 97.11–109.75% at room temperature, 97.29–111.81% at 4 °C, 97.76–109.90% at −70 °C for up to 7 h, and 93.32–99.59% at −70 °C for 196 days. In addition, the mean freeze-thaw stabilities of (S)-(+)-ibuprofen and (R)-(-)-ibuprofen after three freeze-thaw cycles were adequate (92.97–113.38%). In addition, the (S)-(+)-ibuprofen and (R)-(-)-ibuprofen samples after LLE were stable (91.37–109.11%) in the autosampler at 10 °C for 54 h. Deviations from the nominal concentration were within ±15%. Therefore, (S)-(+)-ibuprofen and (R)-(-)-ibuprofen were regarded as stable in beagle dog plasma under all examined conditions, without any change in concentration.

#### 3.2.6. Dilution Integrity

For plasma samples with initially measured concentrations of (S)-(+)-ibuprofen and (R)-(-)-ibuprofen exceeding the ULOQ (80 µg/mL), a five-fold dilution was performed to quantify (S)-(+)-ibuprofen and (R)-(-)-ibuprofen in the beagle dog plasma samples within the calibration curve range. Dilution integrity was validated with five replicates of plasma samples spiked with (S)-(+)-ibuprofen and (R)-(-)-ibuprofen diluted by a factor of five with blank beagle dog plasma to the initial QC concentrations of 0.3, 30, and 64 µg/mL. Table S1 represents the results of the five-fold dilution validation experiment of (S)-(+)-ibuprofen and (R)-(-)-ibuprofen in beagle dog plasma. The accuracy and precision of diluted concentrations satisfied the acceptance criteria, defined as deviations from nominal concentrations within ±15%, suggesting the validity of the dilution integrity of the bioanalytical method for samples with concentrations above the ULOQ.

### 3.3. Application to a Pharmacokinetic Study

**Figure 4:**
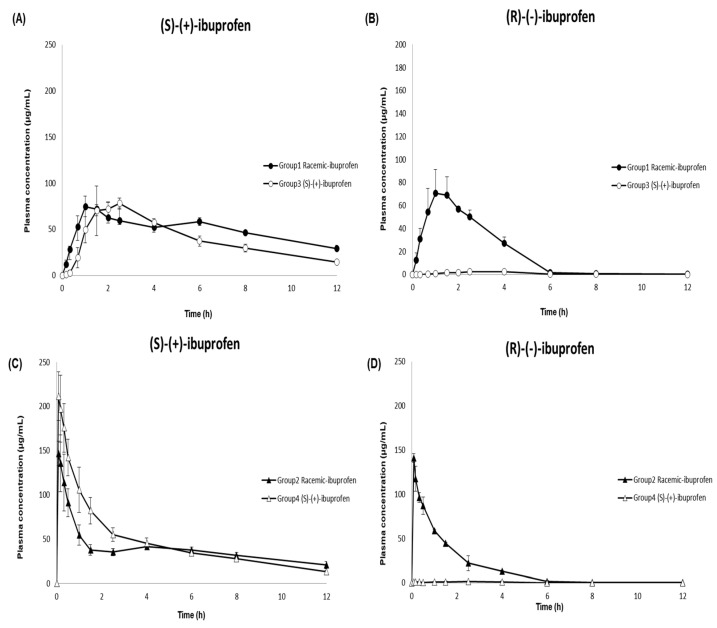
Mean (±SD) plasma concentration–time profile of (S)-(+) ibuprofen (panels (**A**,**C**)) and (R)-(-) ibuprofen (panels (**B**,**D**)) in beagle dogs after the oral administration of 400 mg racemic ibuprofen tablet (●; Group 1) or 300 mg (S)-(+)-ibuprofen tablet (○; Group 3) (panels (**A**,**B**)) and after the intravenous administration of 400 mg racemic ibuprofen solution (▲; Group 2) or 300 mg (S)-(+)-ibuprofen solution (△; Group 4) (panels (**C**,**D**)).

Dexibuprofen ((S)-(+)-ibuprofen), an active enantiomer of ibuprofen, is available as an oral tablet in the market [9,10,46]. In this study, the developed and validated LC–MS/MS method was successfully implemented in a pharmacokinetic study to analyze 144 plasma samples from beagle dogs after oral and intravenous administration of racemic ibuprofen and (S)-(+)-ibuprofen. The mean ± SD plasma concentration–time profiles of (S)-(+)-ibuprofen and (R)-(-)-ibuprofen in the beagle dogs are shown in Figure 4. Table 6 summarizes the pharmacokinetic parameters of racemic ibuprofen and (S)-(+)-ibuprofen. Non-compartmental analyses were performed to calculate the following pharmacokinetic parameters using the noncompartmental methods (WinNonlin version 8.1; Pharsight Corporation, Mountain View, CA, USA): extrapolated AUC_inf_, AUC_last_, *C*_max_, *T*_max_, t_1/2_, CL/F, V_z_/F, and MRT.

Consistent with previous studies evaluating ibuprofen pharmacokinetics after intravenous and oral administration of racemic ibuprofen in humans [47,48,49], the *C*_max_ values of (S)-(+)-ibuprofen after intravenous administration of racemic ibuprofen and (S)-(+)-ibuprofen were 1.98 and 2.50 times higher, respectively, than those after oral administration. The *T*_max_ values were 0.11 and 0.08 h after intravenous administration of racemic ibuprofen and (S)-(+)-ibuprofen, respectively; these values were 1.50 and 2.17 h after oral administration of racemic-ibuprofen and (S)-(+)-ibuprofen, respectively. The AUC_last_ values of (S)-(+)-ibuprofen were comparable between the two different routes of administration (oral: racemic ibuprofen 467.78 and (S)-(+)-ibuprofen 588.14 μg·h/mL; intravenous: racemic ibuprofen 461.71 and (S)-(+)-ibuprofen 557.58 μg· h/mL). The *C*_max_ values of (S)-(+)-ibuprofen after both oral and intravenous administrations of 300 mg (S)-(+)-ibuprofen (oral: 84.57 μg/mL; intravenous: 211.52 μg/mL) were higher than those after administering 400 mg racemic ibuprofen (oral: 77.76 μg/mL; intravenous:153.91 μg/mL). Meanwhile, the *C*_max_ of (S)-(+)-ibuprofen only increased about 1.1 times after oral administration of (S)-(+)-ibuprofen compared to racemic ibuprofen, and the *C*_max_ of (S)-(+)-ibuprofen after intravenous administration of (S)-(+)-ibuprofen increased about 1.5 times higher compared to racemic ibuprofen. Previous studies have reported comparable *C*_max_ values of (S)-(+)-ibuprofen and (R)-(-)-ibuprofen in human plasma after oral administration of racemic ibuprofen [26,39,41,50]. The current study consistently demonstrated similar *C*_max_ values of (S)-(+)-ibuprofen and (R)-(-)-ibuprofen (77.76 ± 9.80 and 74.74 ± 20.02 µg/mL, respectively) in beagle dogs. However, AUC_last_ was larger for (S)-(+)-ibuprofen than for (R)-(-)-ibuprofen after oral administration of racemic ibuprofen in the present study, consistent with previous studies in humans [26,29,39,40,50,51] and dogs [52]. This may have resulted from isomeric inversion from inactive (R)-(-)-ibuprofen to active (S)-(+)-ibuprofen throughout the body [6,12,13,18]. In addition, substantially different pharmacokinetic profiles between (S)-(+)-ibuprofen and (R)-(-)-ibuprofen, particularly slower clearance for (S)-(+)-ibuprofen compared to that for (R)-(-)-ibuprofen (0.60 ± 0.09 vs. 2.21 ± 0.16 L/h), might contribute to the larger AUC_last_ for (S)-(+)-ibuprofen compared to (R)-(-)-ibuprofen. This inference is consistent with a previous study that administered intravenous (S)-(+)-ibuprofen to beagles, suggesting two times higher clearance for (R)-(-)-ibuprofen than that for (S)-(+)-ibuprofen and two times longer MRT for (S)-(+)-ibuprofen than that for (R)-(-)-ibuprofen [53].

Compared to previous studies, the enantiomeric ratio of AUC_(S)-(+)-ibuprofen_/AUC_(R)-(-)-ibuprofen_ estimated in our current study was smaller (2.09 after oral and 2.55 after intravenous administration vs. 3.27 to 6.00 in previous studies) [29,40,54]. The degree of R-to-S inversion may vary depending on the dosage form [51,54] and study subject species; the rate of isomeric inversion may vary in different species with different mechanisms of isomeric inversion, as well as with different routes of administration [55]. Similar to the inference of our current study, Frihmat et al. suggested a lower isomeric inversion ratio after oral administration than after intravenous administration of racemic ibuprofen at the same dose (35.34% vs. 43.62%) in beagle dogs [52]. Overall, these results suggest a lower isomeric inversion rate with oral administration than with intravenous administration [49]. Consistent with previous studies in humans [26,48,50] and dogs [52,53], (R)-(-)-ibuprofen was not detected in the plasma after oral and intravenous administration of (S)-(+)-ibuprofen, suggesting that there was no significant isomeric inversion from (S)-(+)-ibuprofen to (R)-(-)-ibuprofen. Although previous research has suggested bioequivalence between (S)-(+)- and racemic ibuprofen tablets after oral administration of 400 mg to humans [26], no pharmacokinetic or bioequivalence evaluation has been performed for (S)-(+)-ibuprofen after intravenous injections of (S)-(+)- and racemic ibuprofen. Our present study exhibited higher *C*_max_, AUC_last_, and AUC_inf_ of (S)-(+)-ibuprofen after intravenous administration of 300 mg (S)-(+)-ibuprofen than after that of 400 mg racemic ibuprofen, suggesting a potentially higher pharmacological activity of the newly developed (S)-(+)-ibuprofen injection than the racemic ibuprofen injection.

### 3.4. Incurred Sample Reanalysis (ISR)

ISR was executed to determine the reproducibility of the developed analytical method in the present study. Conforming to regulatory guidelines [44,45], the bioanalytical assay was regarded as reproducible if at least 67% of the tested samples had a deviation within ±20% between the original measurement and the reanalysis results. Two of the fifteen ISR samples exceeding the ULOQ were further diluted and reanalyzed as described in Section 3.2.6. All the reanalyzed samples (*n* = 15) satisfied the pre-described regulatory ISR acceptance criteria [56] for both (S)-(+)-ibuprofen and (R)-(-)-ibuprofen (Table S2).

## 4. Conclusions

In this study, a selective and reproducible analytical method was developed and validated to determine the concentration of ibuprofen enantiomers in beagle dog plasma. The developed method was fully validated according to the MFDS and USFDA guidelines [44,45], including the specificity, reliability, and reproducibility of the established quantitation method over the concentration range. In addition, our newly developed method was successfully applied to pharmacokinetic research for the simultaneous quantification of ibuprofen enantiomers after oral and intravenous administration of (S)-(+)-ibuprofen and racemic ibuprofen in beagle dogs (tablets for oral administration, injection solutions for intravenous administration). Based on the observed differences in plasma concentrations of (S)-(+)-ibuprofen following oral or intravenous administration of (S)-(+)-ibuprofen and racemic ibuprofen, the pharmacokinetic profiles of the two enantiomers were substantially different. The unidirectional inversion of R-(-)-ibuprofen to (S)-(+)-ibuprofen after the administration of racemic ibuprofen might contribute to the pharmacokinetic differences between the two enantiomers. Therefore, our findings may assist in understanding the pharmacokinetic properties of ibuprofen after oral and intravenous administration to support further clinical development of (S)-(+)-ibuprofen intravenous injection solutions.

## Figures and Tables

**Table 1:** Retention time, ion transitions for mass spectrometric detection, and mass spectrometry parameters of (S)-(+)-ibuprofen, (R)-(-)-ibuprofen, (S)-(+)-ibuprofen-d3, and (S)-(+)-ketoprofen.

Compounds	Ion Transition (*m*/*z*)	DP (V)	EP (V)	CE (V)	CXP (V)	RT (min)
(S)-(+)-Ibuprofen	205.1 → 160.9	−62.0	−10.0	−12.0	−9.0	10.38
(R)-(-)-Ibuprofen	205.1 → 160.9	−62.0	−8.0	−10.0	−9.0	9.44
(S)-(+)-Ibuprofen-d3	208.1 → 163.9	−62.0	−10.0	−10.0	−9.0	10.31
(S)-(+)-Ketoprofen	253.1 → 208.9	−70.0	−10.0	−12.0	−13.0	8.91

DP: declustering potential, EP: entrance potential, CE: collision energy, CXP: cell exit potential, RT: retention time.

**Table 2:** Linearity of calibration curves after regression analysis for the analytical method to determine (S)-(+)- and (R)-(-)-ibuprofen in beagle dog plasma samples.

Compounds	Number	Slope	Intercept	*r*	*r* ^2^
(S)-(+)-Ibuprofen	1	0.154	−0.001340	0.9995	0.9990
	2	0.155	−0.001220	0.9990	0.9980
	3	0.158	−0.000825	0.9989	0.9978
	4	0.150	−0.000686	0.9985	0.9970
	5	0.145	0.000687	0.9988	0.9976
(R)-(-)-Ibuprofen	1	0.0419	−0.000404	0.9984	0.9968
	2	0.0410	−0.000674	0.9966	0.9932
	3	0.0456	−0.000104	0.9971	0.9942
	4	0.0479	−0.000874	0.9970	0.9940
	5	0.0458	0.000545	0.9959	0.9918

**Table 3:** Intra- and inter-day precision and accuracy of (S)-(+)-ibuprofen and (R)-(-)-ibuprofen (*n* = 5).

Compounds	Nominal Concentration (µg/mL)	Intra-Day (*n* = 5)			Inter-Day (*n* = 15)		
		Mean ± SD (µg/mL)	Precision (CV, %) ^a^	Accuracy (%) ^b^	Mean ± SD (µg/mL)	Precision (CV, %) ^a^	Accuracy (%) ^b^
(S)-(+)-Ibuprofen	0.1	0.10 ± 0.01	7.31	103.60	0.10 ± 0.01	6.60	103.00
	0.3	0.31 ± 0.01	2.03	103.60	0.31 ± 0.01	3.65	101.84
	30	28.96 ± 0.42	1.46	96.53	28.93 ± 0.37	1.28	96.45
	64	66.13 ± 0.35	0.53	103.33	65.04 ± 1.20	1.84	101.63
(R)-(-)-Ibuprofen	0.1	0.10 ± 0.01	7.19	97.60	0.10 ± 0.00	4.87	99.07
	0.3	0.30 ± 0.01	1.75	99.80	0.30 ± 0.01	3.04	100.49
	30	31.16 ± 0.15	0.48	103.86	32.27 ± 0.63	1.93	107.58
	64	69.45 ± 0.67	0.96	108.51	70.98 ± 0.74	1.04	110.90

^a^ CV (%) = (standard deviation of the calculated concentrations/mean concentration) × 100. ^b^ Accuracy (%) = (predicted concentration/nominal concentration) × 100.

**Table 4:** Recovery and matrix effect of (S)-(+)-ibuprofen, (R)-(-)-ibuprofen, (S)-(+)-ibuprofen-d3, and (S)-(+)-ketoprofen (*n* = 6).

Compounds	Nominal Concentration (µg/mL)	Recovery (%)		Matrix Effect (%)	
		Mean ± SD (%)	CV (%)	Mean ± SD (%)	CV (%)
(S)-(+)-Ibuprofen	0.3	85.28 ± 4.73	5.54	93.79 ± 3.89	4.15
	30	82.23 ± 1.44	1.75	86.74 ± 1.95	2.25
	64	82.94 ± 2.29	2.76	89.54 ± 2.06	2.30
	Mean	83.48 ± 2.82	3.35	90.02 ± 2.63	2.90
(R)-(-)-Ibuprofen	0.3	87.89 ± 2.59	2.94	100.99 ± 1.71	1.69
	30	85.02 ± 2.53	2.97	94.38 ± 3.14	3.33
	64	84.01 ± 2.22	2.65	94.63 ± 2.54	2.69
	Mean	85.64 ± 2.45	2.85	96.67 ± 2.46	2.57
(S)-(+)-Ibuprofen-d3	10	87.49 ± 2.94	3.36	87.48 ± 1.72	1.96
(S)-(+)-Ketoprofen	10	84.56 ± 1.12	1.32	92.21 ± 0.78	0.84

**Table 5:** Stability for (S)-(+)-ibuprofen and (R)-(-)-ibuprofen in methanol and beagle dog plasma samples (*n* = 3).

Nominal Concentration (µg/mL)	Solution Stability (Mean ± SD, %)		Plasma Stability (Mean ± SD, %)					
	Stock Room Temperature (3 h)	Working Room Temperature (7 h)	Room Temperature (7 h)	4 °C (7 h)	−70 °C (7 h)	Freeze-Thaw Stability (3 Cycles)	Autosampler (54 h, 10 °C)	Long-Term (−70 °C, 196 Days)
(S)-(+)-Ibuprofen								
0.3	99.04 ± 3.40	98.83 ± 4.07	101.78 ± 0.01	100.56 ± 0.01	99.33 ± 0.01	98.00 ± 0.01	97.22 ± 0.00	99.59 ± 4.21
30	-	-	97.11 ± 0.43	97.29 ± 0.47	97.76 ± 0.51	92.97 ± 0.24	91.37 ± 0.05	93.32 ± 0.21
64	100.15 ± 2.15	99.55 ± 2.64	100.69 ± 0.78	98.77 ± 0.46	99.250 ± 0.65	99.61 ± 0.82	98.48 ± 0.30	97.24 ± 2.53
(R)-(-)-Ibuprofen								
0.3	100.72 ± 3.34	101.73 ± 7.57	97.78 ± 0.00	98.33 ± 0.00	100.22 ± 0.01	95.22 ± 0.00	99.33 ± 0.01	96.36 ± 5.34
30	-	-	108.67 ± 0.21	109.47 ± 0.20	109.90 ± 0.26	104.70 ± 0.21	108.44 ± 0.18	98.05 ± 0.47
64	99.84 ± 1.76	99.57 ± 1.51	109.75 ± 1.08	111.81 ± 0.44	109.74 ± 1.53	113.38 ± 1.29	109.11 ± 0.90	94.87 ± 0.63

**Table 6:** Pharmacokinetic parameter estimates of (S)-(+)-ibuprofen and (R)-(-)-ibuprofen after the oral and intravenous administration of racemic ibuprofen and (S)-(+)-ibuprofen.

Parameter	Oral			
	(S)-(+)-Ibuprofen (Mean ± SD)		(R)-(-)-Ibuprofen (Mean ± SD)	
	Group 1 (Mean ± SD)	Group 3 (Mean ± SD)	Group 1 (Mean ± SD)	Group 3 (Mean ± SD)
*C*_max_ (µg/mL)	77.76 ± 9.80	84.57 ± 12.30	74.74 ± 20.02	- ^a^
AUC_last_ (µg·h/mL)	467.78 ± 20.59	588.14 ± 37.12	223.48 ± 37.04	-
AUC_inf_ (µg·h/mL)	555.65 ± 43.78	847.34 ± 52.17	224.06 ± 37.22	-
*T*_max_ (h)	1.50 ± 0.50	2.17 ± 0.58	1.50 ± 0.50	-
t_1/2_ (h)	6.12 ± 1.44	4.16 ± 0.54	2.07 ± 0.18	-
CL/F(L/h)	0.47 ± 0.03	0.54 ± 0.04	1.82 ± 0.33	-
V_z_/F(L)	4.14 ± 0.75	3.23 ± 0.22	5.49 ± 1.51	-
**Parameter**	**Intravenous**			
	**(S)-(+)-Ibuprofen** **(Mean ± SD)**		**(R)-(-)-Ibuprofen** **(Mean ± SD)**	
	**Group 2** **(Mean ± SD)**	**Group 4** **(Mean ± SD)**	**Group 2** **(Mean ± SD)**	**Group 4** **(Mean ± SD)**
*C*_max_ (µg/mL)	153.91 ± 18.22	211.52 ± 27.52	140.96 ± 4.71	- ^a^
AUC_last_ (µg·h/mL)	461.71 ± 9.42	557.58 ± 66.76	181.28 ± 13.53	-
AUC_inf_ (µg·h/mL)	677.43 ± 98.12	640.78 ± 63.27	181.70 ± 13.36	-
*T*_max_ (h)	0.11 ± 0.05	0.08 ± 0.00	0.08 ± 0.00	-
t_1/2_ (h)	7.06 ± 2.20	4.45 ± 0.80	1.81 ± 0.52	-
CL(L/h)	0.60 ± 0.09	0.47 ± 0.05	2.21 ± 0.16	-
V_z_(L)	5.92 ± 1.03	3.04 ± 0.74	5.74 ± 1.53	-
MRT_last_ (h)	4.77 ± 0.43	3.68 ± 0.23	1.56 ± 0.04	-

^a^: (R)-(-)-ibuprofen was detected. Group 1: oral administration of 400 mg racemic ibuprofen tablet; Group 3: oral administration of 300 mg (S)-(+)-ibuprofen tablet; Group 2: intravenous administration of 400 mg racemic ibuprofen injection; Group 4: intravenous administration of 300 mg (S)-(+)-ibuprofen injection.

## Data Availability

The data presented in this study are available upon request.

## Supplementary Materials

The following supporting information can be downloaded at https://www.mdpi.com/article/10.3390/pharmaceutics15030824/s1, Table S1: Results of 5-fold dilution integrity experiment of (S)-(+)-ibuprofen and (R)-(-)-ibuprofen in beagle dog plasma (*n* = 5); Table S2: Incurred sample reanalysis (ISR) result summary of (S)-(+)-ibuprofen and (R)-(-)-ibuprofen in beagle dogs.

## References

[1] Bushra R., Aslam N. (2010). An overview of clinical pharmacology of ibuprofen. Oman Med. Med. J..

[2] Busson M. (1986). Update on ibuprofen. J. Int. Med. Res..

[3] Davies N.M. (1998). Clinical pharmacokinetics of ibuprofen. Clin. Pharmacokinet..

[4] Kantor T.G. (1979). Ibuprofen. Ann. Intern. Med..

[5] Evans A.M. (1996). Pharmacodynamics and pharmacokinetics of the profens: Enantioselectivity, clinical implications, and special reference to S (+)-ibuprofen. J. Clin. Pharmacol..

[6] Evans A.M. (2001). Comparative pharmacology of S (+)-ibuprofen and (RS)-ibuprofen. Clin. Rheumatol..

[7] Phleps W. (2001). Overview on clinical data of dexibuprofen. Clin. Rheumatol..

[8] Leising G., Resel R., Stelzer F., Tasch S., Lanziner A., Hantich G. (1996). Physical aspects of dexibuprofen and racemic ibuprofen. J. Clin. Pharmacol..

[9] Gliszczyńska A., Sánchez-López E. (2021). Dexibuprofen Therapeutic Advances: Prodrugs and Nanotechnological Formulations. Pharmaceutics.

[10] Kaehler S.T., Phleps W., Hesse E. (2003). Dexibuprofen: Pharmacology, therapeutic uses and safety. Inflammopharmacology.

[11] Lee E.J., Williams K.M., Day R., Graham G., Champion D. (1985). Stereoselective disposition of ibuprofen enantiomers in man. Br. J. Clin. Pharmacol..

[12] Kaiser D.G., Vangiessen G.J., Reischer R.J., Wechter W.J. (1976). Isomeric inversion of ibuprofen (R)-enantiomer in humans. J. Pharm. Sci..

[13] Knihinicki R.D., Williams K.M., Day R.O. (1989). Chiral inversion of 2-arylpropionic acid non-steroidal anti-inflammatory drugs—1: In vitro studies of ibuprofen and flurbiprofen. Biochem. Pharmacol..

[14] Rudy A.C., Knight P.M., Brater D.C., Hall S.D. (1991). Stereoselective metabolism of ibuprofen in humans: Administration of R-, S-and racemic ibuprofen. J. Pharmacol. Exp. Ther..

[15] Hao H., Wang G., Sun J. (2005). Enantioselective pharmacokinetics of ibuprofen and involved mechanisms. Drug Metab. Rev..

[16] Hamman M.A., Thompson G.A., Hall S.D. (1997). Regioselective and stereoselective metabolism of ibuprofen by human cytochrome P450 2C. Biochem. Pharmacol..

[17] López-Rodríguez R., Novalbos J., Gallego-Sandín S., Román-Martínez M., Torrado J., Gisbert J.P., Abad-Santos F. (2008). Influence of CYP2C8 and CYP2C9 polymorphisms on pharmacokinetic and pharmacodynamic parameters of racemic and enantiomeric forms of ibuprofen in healthy volunteers. Pharmacol. Res..

[18] Gabard B., Nirnberger G., Schiel H., Mascher H., Kikuta C., Mayer J.M. (1995). Comparison of the bioavailability of dexibuprofen administered alone or as part of racemic ibuprofen. Eur. J. Clin. Pharmacol..

[19] Bookstaver P.B., Miller A.D., Rudisill C.N., Norris L.B. (2010). Intravenous ibuprofen: The first injectable product for the treatment of pain and fever. J. Pain Res..

[20] Wang P., Qi M., Liu L., Fang L. (2005). Determination of ibuprofen in dog plasma by liquid chromatography and application in pharmacokinetic studies of an ibuprofen prodrug in dogs. J. Pharm. Biomed. Anal..

[21] Jonkman J., Schoenmaker R., Holtkamp A.H., Hempenius J. (1985). Determination of ibuprofen in human plasma by solid phase extraction and reversed-phase high-performance liquid chromatography. J. Pharm. Biomed. Anal..

[22] Zheng C., Hao H., Wang G., Sang G., Sun J., Li P., Li J. (2008). Chiral separation of ibuprofen and chiral pharmacokinetics in healthy Chinese volunteers. Eur. J. Drug Metab. Pharmacokinet..

[23] Naidong W., Lee J.W. (1994). Development and validation of a liquid chromatographic method for the quantitation of ibuprofen enantiomers in human plasma. J. Pharm. Biomed. Anal..

[24] Pettersson K., Olsson A. (1991). Liquid chromatographic determination of the enantiomers of ibuprofen in plasma using a chiral AGP column. J. Chromatogr. B Biomed. Sci. Appl..

[25] Teng X.W., Wang S.W., Davies N.M. (2003). Stereospecific high-performance liquid chromatographic analysis of ibuprofen in rat serum. J. Chromatogr. B.

[26] Wright M.R., Sattari S., Brocks D.R., Jamali F. (1992). Improved high-performance liquid chromatographic assay method for the enantiomers of ibuprofen. J. Chromatogr. B Biomed. Sci. Appl..

[27] Ahn H., Shiu G.K., Trafton W.F., Doyle T.D. (1994). Resolution of the enantiomers of ibuprofen; comparison study of diastereomeric method and chiral stationary phase method. J. Chromatogr. B Biomed. Sci. Appl..

[28] Lemko C.H., Caillé G., Foster R.T. (1993). Stereospecific high-performance liquid chromatographic assay of ibuprofen: Improved sensitivity and sample processing efficiency. J. Chromatogr. B Biomed. Sci. Appl..

[29] Tan S.C., Jackson S., Swift C.G., Hutt A.J. (1997). Enantiospecific analysis of ibuprofen by high performance liquid chromatography: Determination of free and total drug enantiomer concentrations in serum and urine. Chromatographia.

[30] Canaparo R., Muntoni E., Zara G.P., Della Pepa C., Berno E., Costa M., Eandi M. (2000). Determination of Ibuprofen in human plasma by high-performance liquid chromatography: Validation and application in pharmacokinetic study. Biomed. Chromatogr..

[31] Zambakjian C., Sakur A.A. (2020). A new gas chromatographic method development and validation for the simultaneous determination of ibuprofen and caffeine in bulk and pharmaceutical dosage form. Future J. Pharm. Sci..

[32] Way B.A., Wilhite T.R., Smith C.H., Landt M. (1997). Measurement of plasma ibuprofen by gas chromatography-mass spectrometry. J. Clin. Lab. Anal..

[33] Zhao M., Peter C., Holtz M., Hugenell N., Koffel J., Jung L. (1994). Gas chromatographic-mass spectrometric determination of ibuprofen enantiomers in human plasma using R (−)-2, 2, 2-trifluoro-1-(9-anthryl) ethanol as derivatizing reagent. J. Chromatogr. B Biomed. Sci. Appl..

[34] Yilmaz B., Erdem A.F. (2014). Determination of ibuprofen in human plasma and urine by gas chromatography/mass spectrometry. J. AOAC Int..

[35] Aucella F., Lauriola V., Vecchione G., Tiscia G.L., Grandone E. (2013). Liquid chromatography-tandem mass spectrometry method as the golden standard for therapeutic drug monitoring in renal transplant. J. Pharm. Biomed. Anal..

[36] Jemal M. (2000). High-throughput quantitative bioanalysis by LC/MS/MS. Biomed. Chromatogr..

[37] Jemal M., Ouyang Z., Xia Y. (2010). Systematic LC-MS/MS bioanalytical method development that incorporates plasma phospholipids risk avoidance, usage of incurred sample and well thought-out chromatography. Biomed. Chromatogr..

[38] Chen T., Li Q., Lu J., Yu C., Chen C., Li Z. (2016). Determination of ibuprofen enantiomers in human plasma by HPLC–MS/MS: Validation and application in neonates. Bioanalysis.

[39] Sharma P., Guttikar S., Solanki G., Patel D.P., Shrivastav P.S. (2012). Determination of (S)-(+)-and (R)-(-)-ibuprofen enantiomers in human plasma after chiral precolumn derivatization by reversed-phase LC–ESI-MS/MS. Bioanalysis.

[40] Cardoso J.L.C., Lanchote V.L., Pereira M.P.M., de Moraes N.V., Lepera J.S. (2014). Analysis of ibuprofen enantiomers in rat plasma by liquid chromatography with tandem mass spectrometry. J. Sep. Sci..

[41] Nakov N., Petkovska R., Ugrinova L., Kavrakovski Z., Dimitrovska A., Svinarov D. (2015). Critical development by design of a rugged HPLC-MS/MS method for direct determination of ibuprofen enantiomers in human plasma. J. Chromatogr. B.

[42] Bonato P.S., Del Lama M.P.F.M., de Carvalho R. (2003). Enantioselective determination of ibuprofen in plasma by high-performance liquid chromatography–electrospray mass spectrometry. J. Chromatogr. B.

[43] Szeitz A., Edginton A.N., Peng H.T., Cheung B., Riggs K.W. (2010). A validated enantioselective assay for the determination of ibuprofen in human plasma using ultra performance liquid chromatography with tandem mass spectrometry (UPLC-MS/MS). Am. J. Anal. Chem..

[44] Food and Drug Administration (2018). Bioanalytical Method Validation Guidance for Industry. US Department of Health and Human Services. https://www.fda.gov/media/70858/download.

[45] Ministry of Food and Drug Safety (2013). Guideline on Bioanalytical Method Validation. https://www.mfds.go.kr/brd/m210/down.do?brd_id=data0010&seq=13054data_tp=A&file_seq=1.

[46] Yi H.G., Chi M.H., Kim Y., Woo J.S., Park E. (2008). Formulation of a extended release tablet containing dexibuprofen. Arch. Pharm. Res..

[47] Pavliv L., Voss B., Rock A. (2011). Pharmacokinetics, safety, and tolerability of a rapid infusion of iv ibuprofen in healthy adults. Am. J. Health Syst. Pharm..

[48] Cheng H., Rogers J.D., Demetriades J.L., Holland S.D., Seibold J.R., Depuy E. (1994). Pharmacokinetics and bioinversion of ibuprofen enantiomers in humans. Pharm. Res..

[49] Smith H.S., Voss B. (2012). Pharmacokinetics of intravenous ibuprofen. Drugs.

[50] Geisslinger G., Schuster O., Stock K., Loew D., Bach G.L., Brune K. (1990). Pharmacokinetics of S (+)-and R (−)-ibuprofen in volunteers and first clinical experience of S (+)-ibuprofen in rheumatoid arthritis. Eur. J. Clin. Pharmacol..

[51] Jamali F., Singh N.N., Pasutto F.M., Russell A.S., Coutts R.T. (1988). Pharmacokinetics of ibuprofen enantiomers in humans following oral administration of tablets with different absorption rates. Pharm. Res..

[52] Frihmat R., Cardot J., Beyssac E., Boucher M., Aiache J. (2000). Bioinversion of ibuprofen enantiomers after administration in dogs: Estimation of a novel index. Eur. J. Drug Metab. Pharmacokinet..

[53] Beck W.S., Geisslinger G., Engler H., Brune K. (1991). Pharmacokinetics of ibuprofen enantiomers in dogs. Chirality.

[54] Sattari S., Jamali F. (1994). Evidence of absorption rate dependency of ibuprofen inversion in the rat. Chirality.

[55] Wsol V., Skálová L., Szotáková B. (2004). Chiral inversion of drugs: Coincidence or principle?. Curr. Drug Metab..

[56] De Boer T., Wieling J. (2011). Incurred sample accuracy assessment: Design of experiments based on standard addition. Bioanalysis.

