# Supplementary material for: A Validated Chiral LC–MS/MS Method for the Enantioselective Determination of (S)-(+)- and (R)-(-)-Ibuprofen in Dog Plasma: Its Application to a Pharmacokinetic Study
Source: Pharmaceutics. 2023 Mar 2;15(3):824. doi: 10.3390/pharmaceutics15030824 (PMC10051808; doi:10.3390/pharmaceutics15030824)
Supplement: Supplementary file 1 [file pharmaceutics-15-00824-s001.zip › pharmaceutics-2079313-supplementary.pdf]

SUPPLEMENTARY MATERIAL

A validated chiral LC-MS/MS method for the enantioselective determination of (S)-(+)- and (R)-(-)-ibuprofen in dog plasma: its application to a pharmacokinetic study

Sanghee Choi [1,2], Wang-Seob Shim[2], Jiyoung Yoon[1,2,] Doowon Choi[1,2], Jinseong Lee[3], Soo-Heui Paik[4], Eun-Kyoung Chung[5] and Kyung-Tae Lee[1,2,6*]

[1] Department of Biomedical and Pharmaceutical Sciences, Graduate School, Kyung Hee University, Seoul, 02447, Republic of Korea

[2] Kyung Hee Drug Analysis Center, College of Pharmacy, Medical Center, Kyung Hee University, Seoul, 02447, Republic of Korea

[3] Department of BD&RA Division, BNC KOREA Inc, Seoul, 06296, Republic of Korea

[5] Department of Pharmacy, College of Pharmacy, Kyung Hee University, Seoul 02447, Republic of Korea

[6] Department of Pharmaceutical Biochemistry, College of Pharmacy, Kyung Hee University, Seoul, 02447, Republic of Korea

Supplementary data

Table S1. Results of 5-fold dilution integrity experiment of (S)-(+)-ibuprofen and (R)-(-)-ibuprofen in beagle dog plasma (n=5).

Compound	Nominal concentration (μg/mL)	Dilution factor 5		
		Mean ± SD, % (μg/mL)	CV (%)	Accuracy (%)
**(S)-(+)-Ibuprofen**	0.3	0.31 ± 0.01	3.52	104.87
	30	30.27 ± 0.46	1.53	100.90
	64	67.54 ± 0.69	1.02	105.54
**(R)-(-)-Ibuprofen**	0.3	0.31 ± 0.02	5.22	102.13
	30	32.23 ± 0.44	1.38	107.42
	64	69.77 ± 2.66	3.81	109.01

Table S2. Incurred sample reanalysis (ISR) result summary of (S)-(+)-ibuprofen and (R)-(-)-ibuprofen in beagle dogs.

	**Selected Sample Numbers**	**Accepted Sample Number**	**Results (%)**
**(S)-(+)-Ibuprofen**	15	15	100.00
**(R)-(-)-Ibuprofen**	15	15	100.00
